# Transcriptomic analysis of sea cucumber (*Holothuria leucospilota*) coelomocytes revealed the echinoderm cytokine response during immune challenge

**DOI:** 10.1186/s12864-020-6698-6

**Published:** 2020-04-16

**Authors:** Xiaofen Wu, Ting Chen, Da Huo, Zonghe Yu, Yao Ruan, Chuhang Cheng, Xiao Jiang, Chunhua Ren

**Affiliations:** 1 CAS Key Laboratory of Tropical Marine Bio-resources and Ecology (LMB), Guangdong Provincial Key Laboratory of Applied Marine Biology (LAMB), South China Sea Institute of Oceanology, Chinese Academy of Sciences, Guangzhou, 510301 PR China; 2 University of Chinese Academy of Sciences, Beijing, 100049 PR China; 3 Institution of South China Sea Ecology and Environmental Engineering, Chinese Academy of Sciences, ISEE, CAS, Guangzhou, PR China; 4 South China Sea Bio-Resource Exploitation and Utilization Collaborative Innovation Center, Guangzhou, 510301 People’s Republic of China

**Keywords:** Sea cucumber, Coelomocytes, Transcriptome, RNA-seq, Differentailly expressed genes, Immune response, Cytokines

## Abstract

**Background:**

The sea cucumber *Holothuria leucospilota* belongs to echinoderm, which is evolutionally the most primitive group of deuterostomes. Sea cucumber has a cavity between its digestive tract and the body wall that is filled with fluid and suspended coelomic cells similar to blood cells. The humoral immune response of the sea cucumber is based on the secretion of various immune factors from coelomocytes into the coelomic cavity. The aim of this study is to lay out a foundation for the immune mechanisms in echinoderms and their origins in chordates by using RNA-seq.

**Results:**

Sea cucumber primary coelomocytes were isolated from healthy *H. leucospilota* and incubated with lipopolysaccharide (LPS, 10 μg/ml), polyinosinic-polycytidylic acid [Poly (I:C), 10 μg/ml] and heat-inactived *Vibrio harveyi* (10^7^ cell/ml) for 24 h, respectively. After high-throughput mRNA sequencing on an Illumina HiSeq2500, a de novo transcriptome was assembled and the Unigenes were annotated. Thirteen differentially expressed genes (DEGs) were selected randomly from our data and subsequently verified by using RT-qPCR. The results of RT-qPCR were consistent with those of the RNA-seq (*R*^2^ = 0.61). The top 10 significantly enriched signaling pathways and immune-related pathways of the common and unique DEGs were screened from the transcriptome data. Twenty-one cytokine candidate DEGs were identified, which belong to 4 cytokine families, namely, BCL/CLL, EPRF1, IL-17 and TSP/TPO. Gene expression in response to LPS dose-increased treatment (0, 10, 20 and 50 μg/ml) showed that IL-17 family cytokines were significantly upregulated after 10 μg/ml LPS challenge for 24 h.

**Conclusion:**

A de novo transcriptome was sequenced and assembled to generate the gene expression profiling across the sea cucumber coelomocytes treated with LPS, Poly (I:C) and *V. harveyi*. The cytokine genes identified in DEGs could be classified into 4 cytokine families, in which the expression of IL-17 family cytokines was most significantly induced after 10 μg/ml LPS challenge for 24 h. Our findings have laid the foundation not only for the research of molecular mechanisms related to the immune response in echinoderms but also for their origins in chordates, particularly in higher vertebrates.

## Background

*Holothuria leucospilota* is a tropical sea cucumber that belongs to phylum Echinodermata, class Holothuroidea, order Aspidochirotida and family Holothuriidae. Naturally, *H. leucospilota* is distributed in the Indo-West Pacific, mostly from eastern Africa to the Hawaii islands and Society islands in the Pacific ocean, and from southern Japan to the Sark bay in Australia [1]. In recent years, *H. leucospilota* has become an emerging aquaculture species in southern China [2]. Evolutionally, echinoderms are positioned taxonomically at the base of deuterostomes, along with the higher-order hemichordate and chordate groups [3]. Therefore, studies on the biological processes in echinoderms, such as development, reproduction, metabolism and immunity, may provide new insights not only for echinoderms themselves, but also for the origins of these biological processes in chordates, particularly in higher vertebrates.

Given that lacking of the adaptive immunity, the innate immunity is the major mechanism for sea cucumber to defend the environmental pathogens. The innate immunity of sea cucumber includes multiple immune-related factors, such as, antimicrobial peptides (e.g. lectins, lysozyme, clotting protein and complement) [4, 5], antimicrobial reactive oxygen species [6], pattern recognition receptors, apoptosis [7, 8] and immune cytokines [9]. Sea cucumber has a cavity between its digestive tract and body wall that is filled with coelomic fluid and suspended coelomocytes that are similar to the hematocyts of vertebrates. In sea cucumber, the cellular immunity is executed by coelomocytes, and the humoral immunity is based on a variety of macromolecules in the coelomic cavity that secreted by coelomocytes [10–12]. When sea cucumbers are infected by pathogenic microbes, they rely on their cellular and humoral immune responses to identify and eliminate the invading microbes and repair the wounds [9].

Next-generation sequencing (NGS) technology is a revolutionary change to traditional sequencing technology. The high-throughput mRNA sequencing (RNA-seq) is a transcriptomic research method that provides information on transcript expression and has the advantages of being easy-handle and low-cost [13]. For non-model organisms, RNA-Seq is not limited to detecting transcripts that correspond to existing genomic sequence. RNA-Seq focus more on the coding region of genes and has very low background signal [14]. Therefore, RNA-Seq is particularly attractive for non-model organisms with genomic sequences that are yet to be determined.

To date, transcriptomic sequencing technology has been widely applied for analyzing the gene expression profiles of echinoderms in a variety of developmental and physiological processes, and under multiple infected- or stressed-conditions. In the sea urchin *Heliocidaris erythrogramma*, RNA-seq has been used to measure the mRNA expression profiles of larvae, metamorphism and post-metamorphism life cycle stages, to elucidate the evolutional and developmental mechanism of the radial adult body in echinoderms [15]. The expression dynamics across development has also been measured by RNA-seq in three sea urchin species: the lecithotroph *H. erythrogramma*, the closely related planktotroph *H. tuberculata*, and an outgroup planktotroph *Lytechinus variegatus*, to reveal how evolutionary changes in gene regulation contribute to phenotypic differences between different species [16]. In addition, RNA-seq has been used to analyze the differentially expressed genes (DEGs) in the body cavity cells between the viral-infected and normal starfish *Pycnopodia helianthoides*, to illustrate the immune and nervous responses to the sea star wasting disease [17]. Furthermore, the next-generation transcriptome data from 42 echinoderm specimens from 24 orders, 37 families have been collected to establish a web-based application (http://echinotol.org) to identify orthologs suitable for phylogenetic analysis in assembling the echinoderm tree of life [18].

In sea cucumber, transcriptomic sequencing has been performed together with genomic and proteomic analyses in *Apostichopus japonicus*, to facilitate the molecular underpinnings of visceral regeneration [19]. RNA-seq has been applied independently to analyze the DEGs between normal and regenerating radial nerve cord in *Holothuria glaberrima*, and revealed the key roles of extracellular matrix (ECM) remodeling and ECM-cell interactions in regeneration [20]. In addition, the applications of transcriptomic analyses in sea cucumber include identification of long noncoding RNA species [21], illustration of the mechanisms of aestivation [22], abnormal development [23], and body wall pigmentation [24]. Moreover, immune-related genes in the *A. japonicus* coelomocytes under *Vibrio splendidus* challenge have been identified, which are clustered into the immune pathways of endocytosis, lysosome, chemokine, and MAPK and ERBB signaling [25]. However, there is still limited research reports on the species and response of the cytokines secreted by coelomocytes of sea cucumber under challenge of immune stimuli.

In this study, high-throughput transcriptomic sequencing and bioinformatics analysis were performed on the primary coelomocytes isolated from the sea cucumber *H. leucospilota* and treated with three different immune stimuli including lipopolysaccharide (LPS), polyinosinic-polycytidylic acid [poly (I:C)] and heat-inactived *Vibrio harveyi* for 24 h. Based on the DEGs between different groups, the immune-related pathways were screened out and the responded cytokines were identified. This study provides evidences for the potential roles of cytokines in the innate immunity of sea cucumber.

## Results

### Illumina draft reads and sequence assembly

**Fig. 1:**
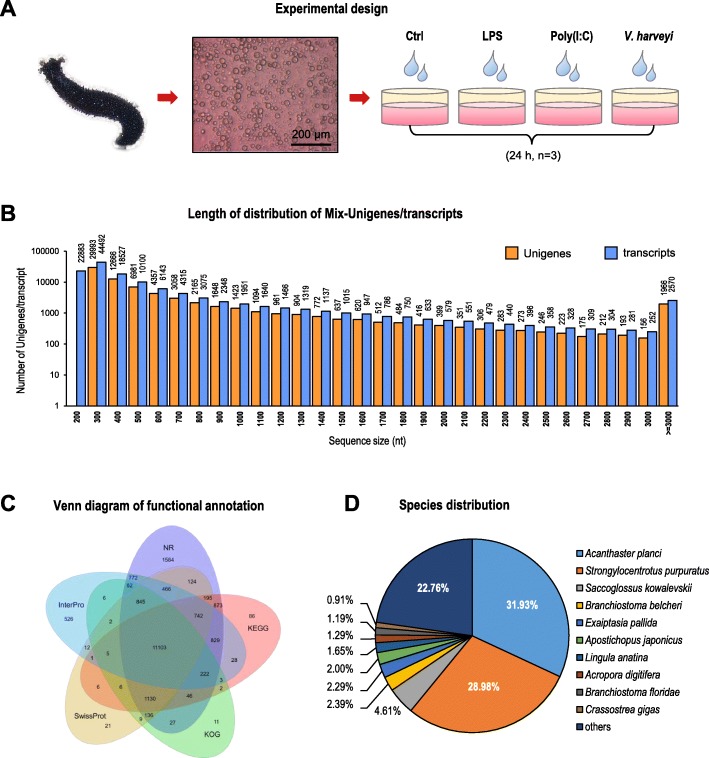
Experimental design and transcriptome information. **a** Experimental design. Sea cucumber coelomocytes isolated from *H. leucospilota were* challenged with LPS (10 μg/ml), Poly (I:C) (10 μg/ml) and *V.harveyi* (10^7^ cell/ml) for 24 h with three biological duplicates. **b** The length distribution of all-Unigene. The X-axis represents the sequence size, and the Y-axis represents the number of Mix-Unigene. The orange bar shows the number of unigene which is the representative sequences, and the blue bar shows the number of transcripts which include the rough sequences. **c** Venn diagram of Unigene annotation. The databases used for gene annotation include NR、KOG、KEGG、SwissProt and InterPro. **d** Species distribution of Unigene annotation in NR database

Primary coelomocytes isolated from the sea cucumber *H. leucospilota* were respectively challenged with LPS, poly (I:C) and heat-inactivated *V. harveyi* for 24 h (Fig. 1a), and twelve cDNA libraries were constructed to perform Illumina sequencing. After assembly and redundancy removal, a transcriptome with twelve RNA-seq libraries in total of 6.69 GB with 73,472 identified Unigenes was obtained and submitted to GenBank under the BioProject accession No. PRJNA559679. The total length, average length, N50, and GC content of the Unigenes were 47,163,631 bp, 641 bp, 1015 bp and 39.54%, respectively. The number of transcripts and Unigenes decreased with increasing of length, and the majority of them were concentrated in 200–3000 bp (Fig. 1b). The Unigene sequences were annotated to seven functional databases, and 20,926 (28.48%) of them were significantly matched to at least one of the databases (Table 1), in which 19,156 (26.07%) to NR, 3990 (5.43%) to NT, 14803 (20.15%) to SwissProt, 13,615 (18.53%) to KOG, 15277 (20.79%) to KEGG, 7097 (9.66%) to GO and 15,624 (21.27%) to InterPro. The annotation results for five databases were further showed in a Venn diagram: 1584, 11, 88, 21 and 528 genes were independently annotated into the NR, KOG, KEGG, SwissProt and InterPro databases, respectively, and the intersection set of these five databases was 11,103 (Fig. 1c). For the NR database, the annotated Unigenes were majorly matched to *Strongylocentrotus purpuratus* (5552, 28.98%), *Acanthaster planci* (6117, 31.93%), *Saccoglossus kowalevskii* (883, 4.61%), *Branchiostoma belcheri* (457, 2.39%) and other species (6147, 32.09%, Fig. 1d).

**Table 1:** Functional annotation analysis

	Total	NR	NT	SwissPprot	KEGG	KOG	InterPro	GO	Intersection	Overall
No. of gene	73,472	19,156	3990	14,803	15,277	13,615	15,624	7097	1285	20,926
Percentage	100%	26.07%	5.43%	20.15%	20.79%	18.53%	21.27%	9.66%	1.75%	28.48%

### Analysis of DEGs and validation of RNA-Seq data by RT-qPCR

**Fig. 2:**
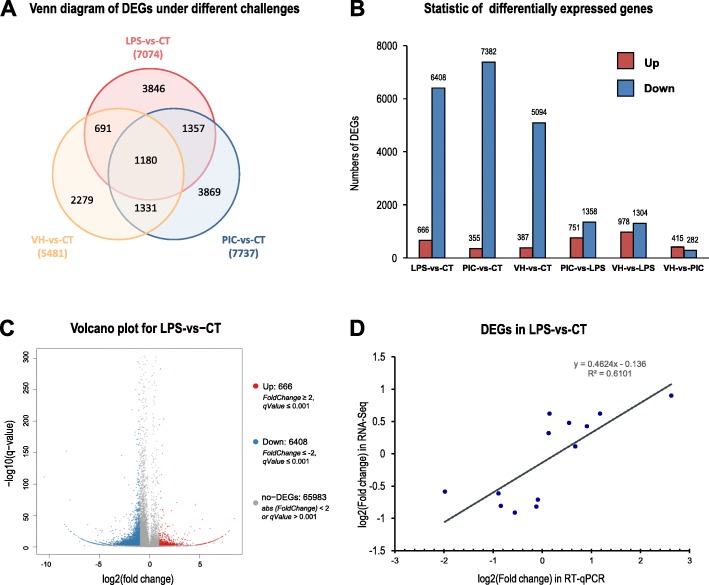
Comparative transcriptome analysis of DEGs among different immune challenges. **a** Venn diagram of unique and common DEGs among the immune challenges of LPS、Poly (I:C) and *V. harveyi*. **b** the number of up- and down-regulated DEGs in each immune challenge group compared with control group (the first 3 combination bar) and the pairwise comparison of the three different immune challenge groups (the last 3 combination bar). **c** Volcano map of DEGs in LPS-vs-CT group: X-axis represents the fold change value after log2 conversion, and Y-axis represents the fold change after -log10 conversion. Red dots represent the up-regulated DEGs, blue dots represent the down-regulated DEGs, and gray dots represents the non-DEGs. **d** comparison results between RNA-Seq by RT-qPCR data in LPS-vs-CT group. X axis shows the fold changes of gene expression in RT-qPCR data, while Y axis represents the fold changes of gene expression in RNA-Seq data

The transcriptomic data obtained from the control group (CT), LPS-treatment group (LPS), Poly (I:C)-treatment group (PIC) and *V. harveyi*-treatment group (VH) were analyzed comparatively. The results showed that the co-expressed DEGs for the three groups were 1180, and the uniquely-expressed genes in the LPS-vs-CT, PIC-vs-CT and VH-vs-CT groups were 3846, 3869 and 2279, respectively (Fig. 2a). The significantly DEGs were acquired by comparing of the gene expression between the LPS, PIC and VH groups and the CT group with the following criteria: *P* ≤ 0.01, |log 2-fold-change| ≥ 1 and false discovery rate (FDR) ≤ 0.05. Finally, 7074 DEGs in the LPS-vs-CT comparison (666 upregulated and 6408 downregulated), 7737 DEGs in the PIC-vs-CT comparison (355 upregulated and 7382 downregulated), and 5481 DEGs in the VH-vs-CT comparison (387 upregulated and 5094 downregulated) were obtained (Fig. 2b).

To validate the gene expression results of the RNA-seq data, 13 significant DEGs from the LPS-vs-CT comparison with the criteria of *P* < 0.01, FDR < 0.05 and fold change of at least 4 were selected for RT-qPCR validation. The volcano plot for the CT-vs-LSP comparison was shown in Fig. 2c. The expression levels of the selected 13 genes in the LPS challenge group were normalized to the control group. As anticipated, the RT-qPCR data showed a positive linear relationship with the RNA-Seq data (Fig. 2d), and there was no statistically significant difference between the two datasets (*P* > 0.05). This result suggested that the RNA-Seq was a positively related reference for expression profiling study on the whole, and the assembly quality of the sequences was desirable.

### Functional classification and enrichment analysis of GO terms and KEGG pathways for co-expressed DEGs

**Fig. 3:**
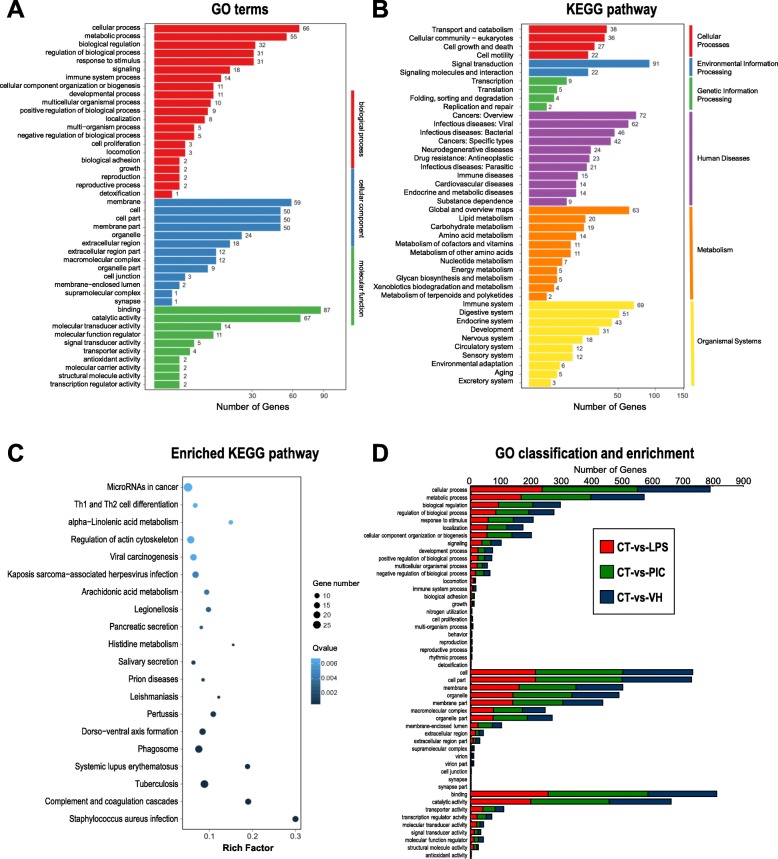
Co-expressed DEGs of functional classification. **a** GO classification of a co-expressed gene. The X-axis represents the enrichment factor value, and the Y-axis represents the path name. **b** Functional classification of KEGG of co-expressed genes. **c** Pathway enrichment distribution of co-expressed genes. The X-axis represents the enrichment factor value, and the Y-axis represents the path name. The color represents q-value, which is corrected *p*-value ranging from 0~1, and less q-value means greater intensiveness. The size of the point represents the number of DEGs (the larger the point, the larger the number; the smaller the point, the smaller the number). Rich Factor refers to the enrichment Factor value, which is the quotient between the foreground value (number of DEGs) of a certain pathway on the annotation and the background value (number of all genes) of a certain pathway on the annotation. The larger the data is, the more obvious the enrichment result will be. **d** GO classification and enrichment of differentially expressed genes under different immune challenges. The Y axis (horizontal direction) represents the number of genes, and the X axis (vertical direction) represents the specific classification under the three functional categories of GO. The red bars represent the GO classification entries annotated by the DEGs in LPS compared to the control group, and accordingly, the purple and blue represent those of Poly (I:C) and *V. harveyi*

The co-expressed DEGs after the three different immune challenges may represent the essential genes for immune defense in sea cucumber. GO (gene ontology) is a major bioinformatics initiative to unify the classification of genes and gene product attributes across all species. Among the total 1180 co-expressed DEGs, 796 were annotated with GO terms, in which 321 were annotated as “biological process”, 279 were annotated as “cellular components” and 196 were annotated as “molecular functions” (Fig. 3a).

KEGG (Kyoto Encyclopedia of Genes and Genomes) is a reference database dealing with genomes, biological pathways, diseases, drugs, and chemical substances. In this case, 1003 co-expressed DEGs were annotated in KEGG, and the number of DEGs in cellular processes, environmental information processing, genetic information processing, human diseases, metabolism and organismal systems categories were 123, 113, 20, 344, 161 and 242, respectively (Fig. 3b).

Gene set enrichment analysis (GSEA, also called functional enrichment analysis) is a method to identify classes of genes or proteins that are overrepresented in a large set of genes or proteins and [26]. The co-expressed DEGs in this study were annotated into 273 KEGG pathways. The top 10 and top 20 significantly enriched pathways were shown in Table 2 and Fig. 3c, respectively. The pathways of *Staphylococcus aureus* infection, complement and coagulation cascades, and tubercuosis were the three most significantly enriched pathways, with a total of 15, 16 and 24 co-expressed DEGs annotation, respectively (Table 2). In addition, the top 10 immune-related KEGG pathways with the highest number of co-expressed genes were shown in Table 3. Within them, the most significant immune-related KEGG pathways were complement and coagulation cascades, which contained 84 annotated genes with 16 co-expressed DEGs. These results laid a foundation for discovering the immune-related genes and developing the immune responding mechanisms of sea cucumber. The functional classification and enrichment analysis of GO terms and KEGG pathways for the DEGs were further performed for the challenges of different immune stimuli (Fig. 3d). In this case, the totals of 2591, 3260 and 2585 Unigenes respectively for the comparisons of the LPS-vs-CT, PIC-vs-CT and VH-vs-CT were annotated in GO terms. Among three different immune challenge groups, the Poly (I:C) treatment group had the most DEGs annotated into the GO terms, indicating that more genes were involved in the administration of Poly (I:C) in the sea cucumber coelomocytes when compared to other two immune stimuli.

**Table 2:** The top 10 KEGG pathways with the highest enrichment of co-expressed DEGs

Pathway ID	DEGs annotation in the pathway	All genes annotation in the pathway	***P***-values	Pathway annotation
**ko05150**	15 (3.85%)	50 (0.33%)	9.73403e-13	*Staphylococcus aureus* infection
**ko04610**	16 (4.1%)	84 (0.55%)	3.141596e-10	Complement and coagulation cascades
**ko05152**	24 (6.15%)	271 (1.77%)	1.258897e-07	Tuberculosis
**ko05322**	10 (2.56%)	53 (0.35%)	7.672128e-07	Systemic lupus erythematosus
**ko04145**	22 (5.64%)	291 (1.9%)	5.889262e-06	Phagosome
**ko04320**	18 (4.62%)	213 (1.39%)	9.237491e-06	Dorso-ventral axis formation
**ko05133**	13 (3.33%)	119 (0.78%)	1.082585e-05	Pertussis
**ko05140**	11 (2.82%)	89 (0.58%)	1.597061e-05	Leishmaniasis
**ko05020**	15 (3.85%)	172 (1.13%)	3.550144e-05	Prion diseases

**Table 3:** The top 10 immune-related KEGG pathways with the highest number of co-expressed DEGs

Pathway ID	DEGs annotation in the pathway	All genes annotation in the pathway	***P***-values	Pathway annotation
**ko04610**	16 (4.1%)	84 (0.55%)	3.141596e-10	Complement and coagulation cascades
**ko04658**	15 (3.85%)	218 (1.43%)	0.0004926241	Th1 and Th2 cell differentiation
**ko04650**	7 (1.79%)	115 (0.75%)	0.02799556	Natural killer cell mediated cytotoxicity
**ko04611**	10 (2.56%)	213 (1.39%)	0.04710404	Platelet activation
**ko04670**	9 (2.31%)	208 (1.36%)	0.08609158	Leukocyte transendothelial migration
**ko04666**	7 (1.79%)	155 (1.01%)	0.102463	Fc gamma R-mediated phagocytosis
**ko04620**	5 (1.28%)	118 (0.77%)	0.1839742	Toll-like receptor signaling pathway
**ko04659**	4 (1.03%)	88 (0.58%)	0.1874437	Th17 cell differentiation
**ko04062**	7 (1.79%)	188 (1.23%)	0.2058452	Chemokine signaling pathway
**ko04657**	5 (1.28%)	127 (0.83%)	0.2245483	IL-17 signaling pathway

### The most significant enrichment pathway of DEGs in different immune challenges

A hypergeometric test was used for enrichment analysis of all signal pathways in the KEGG database. Compared to the control group, the number of DEGs annotated to specific pathways for the LPS, Poly (I:C) and *V. harveyi* treatment groups were 3415, 4261 and 3120, respectively. The top 10 most significantly enriched pathways for each challenge were shown in Table 4, in which the common pathways for the three treatments groups were the ABC transporter pathway, ubiquitin-mediated proteolysis pathway and inositol phosphate metabolism pathway. The glucagon signaling pathway was a common pathway for the Poly (I:C) and *V. harveyi* treatment groups, and other pathways were specific for each treatment. Analysis of the DEGs enriched KEGG pathways provides an effective basis for studying the immune defense process, biological function and metabolic pathways in the sea cucumber coelomocytes.

**Table 4:** The top 10 KEGG pathways with the highest enrichment of DEGs in groups with different immune challenges

Pathway ID	DEGs annotation in the pathway	All genes annotation in the pathway	***P***-values	Pathway annotation
**CT-vs-LPS**				
**ko02010**	40 (3.26%)	163 (1.07%)	1.040587e-10	ABC transporters
**ko04120**	41 (3.34%)	274 (1.79%)	7.710349e-05	Ubiquitin mediated proteolysis
**ko00250**	15 (1.22%)	88 (0.58%)	0.004140363	Alanine, aspartate and glutamate metabolism
**ko05215**	21 (1.71%)	147 (0.96%)	0.006927921	Prostate cancer
**ko00512**	7 (0.57%)	30 (0.2%)	0.008355145	Mucin type O-glycan biosynthesis
**ko04919**	43 (3.5%)	374 (2.45%)	0.01077703	Thyroid hormone signaling pathway
**ko05226**	30 (2.44%)	247 (1.62%)	0.01495566	Gastric cancer
**ko04330**	28 (2.28%)	227 (1.49%)	0.01511225	Notch signaling pathway
**ko04975**	12 (0.98%)	76 (0.5%)	0.01751861	Fat digestion and absorption
**ko00562**	15 (1.22%)	105 (0.69%)	0.0203857	Inositol phosphate metabolism
**CT-vs-PIC**				
**ko00562**	22 (1.45%)	105 (0.69%)	0.0005556445	Inositol phosphate metabolism
**ko04214**	30 (1.98%)	167 (1.09%)	0.0009830988	Apoptosis – fly
**ko04922**	26 (1.71%)	138 (0.9%)	0.001015626	Glucagon signaling pathway
**ko04630**	23 (1.52%)	124 (0.81%)	0.002380328	Jak-STAT signaling pathway
**ko05223**	19 (1.25%)	100 (0.65%)	0.004151803	Non-small cell lung cancer
**ko04070**	24 (1.58%)	142 (0.93%)	0.006645948	Phosphatidylinositol signaling system
**ko00564**	25 (1.65%)	151 (0.99%)	0.007416318	Glycerophospholipid metabolism
**ko00520**	15 (0.99%)	78 (0.51%)	0.009115605	Amino sugar and nucleotide sugar metabolism
**ko05211**	21 (1.38%)	124 (0.81%)	0.01041359	Renal cell carcinoma
**ko04120**	39 (2.57%)	274 (1.79%)	0.0137763	Ubiquitin mediated proteolysis
**CT-vs-VH**				
**ko02010**	24 (2.33%)	163 (1.07%)	0.0002372665	ABC transporters
**ko04922**	20 (1.94%)	138 (0.9%)	0.0009303992	Glucagon signaling pathway
**ko04931**	22 (2.14%)	169 (1.11%)	0.002216142	Insulin resistance
**ko04520**	24 (2.33%)	197 (1.29%)	0.00347834	Adherens junction
**ko04711**	4 (0.39%)	11 (0.07%)	0.004603775	Circadian rhythm – fly
**ko04710**	7 (0.68%)	35 (0.23%)	0.007846637	Circadian rhythm
**ko00562**	14 (1.36%)	105 (0.69%)	0.01068485	Inositol phosphate metabolism
**ko01523**	13 (1.26%)	95 (0.62%)	0.01105092	Antifolate resistance
**ko00534**	10 (0.97%)	65 (0.43%)	0.01116436	Glycosaminoglycan biosynthesis-heparan sulfate / heparin
**ko04962**	10 (0.97%)	65 (0.43%)	0.01116436	Vasopressin-regulated water reabsorption

### Analysis of immune-related pathway in different immune challenges

Compared to the control group, the DEGs of the LPS, Poly (I: C) and *V. harveyi* treatment groups were categorized into 308, 316 and 305 annotated KEGG pathways, respectively. Accordingly, 18, 22 and 23 significantly enriched immune-related pathways for LPS, Poly (I: C) and *V. harveyi* treatments, respectively, were identified according to the immune system of KEGG pathways. The most significantly enriched immune-related pathways for LPS, PIC and *V. harveyi* treatments were the Th1 and Th2 cell differentiation signaling pathways, the intestinal immune network for IgA production pathway, and the Fc gamma R-mediated phagocytosis pathway, respectively. Other top 10 significantly enriched immune-related pathways for the three immune challenges are shown in Table 5. Base on the analysis of different regulatory pathways of the DEGs in KEGG, the mechanism of cellular immune response of the sea cucumber coelomocytes can be understood more directly.

**Table 5:** The top 10 immune-related KEGG pathways with the highest enrichment of DEGs in groups with different immune challenges

Pathway ID	DEGs annotation in the pathway	All genes annotation in the pathway	***P***-values	Pathway annotation
**CT-vs-LPS**				
**ko04658**	21 (1.71%)	218 (1.43%)	0.2218435	Th1 and Th2 cell differentiation
**ko04670**	20 (1.63%)	208 (1.36%)	0.2311792	Leukocyte transendothelial migration
**ko04650**	11 (0.9%)	115 (0.75%)	0.3179508	Natural killer cell mediated cytotoxicity
**ko04666**	14 (1.14%)	155 (1.01%)	0.3636409	Fc gamma R-mediated phagocytosis
**ko04659**	8 (0.65%)	88 (0.58%)	0.4117395	Th17 cell differentiation
**ko05340**	4 (0.33%)	42 (0.27%)	0.4392712	Primary immunodeficiency
**ko04623**	9 (0.73%)	105 (0.69%)	0.4703544	Cytosolic DNA-sensing pathway
**ko04640**	4 (0.33%)	46 (0.3%)	0.5109961	Hematopoietic cell lineage
**ko04664**	8 (0.65%)	98 (0.64%)	0.5344647	Fc epsilon RI signaling pathway
**ko04672**	2 (0.16%)	23 (0.15%)	0.561599	Intestinal immune network for IgA production
**CT-vs-PIC**				
**ko04672**	6 (0.4%)	23 (0.15%)	0.02188151	Intestinal immune network for IgA production
**ko04670**	28 (1.84%)	208 (1.36%)	0.05993948	Leukocyte transendothelial migration
**ko04650**	17 (1.12%)	115 (0.75%)	0.06204072	Natural killer cell mediated cytotoxicity
**ko04666**	21 (1.38%)	155 (1.01%)	0.08842557	Fc gamma R-mediated phagocytosis
**ko04664**	13 (0.86%)	98 (0.64%)	0.1727241	Fc epsilon RI signaling pathway
**ko04623**	13 (0.86%)	105 (0.69%)	0.2419115	Cytosolic DNA-sensing pathway
**ko04640**	6 (0.4%)	46 (0.3%)	0.3049254	Hematopoietic cell lineage
**ko04662**	12 (0.79%)	102 (0.67%)	0.312857	B cell receptor signaling pathway
**ko04620**	13 (0.86%)	118 (0.77%)	0.3906605	Toll-like receptor signaling pathway
**ko05340**	5 (0.33%)	42 (0.27%)	0.4066475	Primary immunodeficiency
**CT-vs-VH**				
**ko04666**	17 (1.65%)	155 (1.01%)	0.03195124	Fc gamma R-mediated phagocytosis
**ko04062**	18 (1.75%)	188 (1.23%)	0.08324536	Chemokine signaling pathway
**ko05340**	5 (0.49%)	42 (0.27%)	0.1499922	Primary immunodeficiency
**ko04650**	11 (1.07%)	115 (0.75%)	0.1515229	Natural killer cell mediated cytotoxicity
**ko04672**	3 (0.29%)	23 (0.15%)	0.1992882	Intestinal immune network for IgA production
**ko04664**	9 (0.87%)	98 (0.64%)	0.2139022	Fc epsilon RI signaling pathway
**ko04659**	8 (0.78%)	88 (0.58%)	0.2403939	Th17 cell differentiation
**ko05321**	2 (0.19%)	14 (0.09%)	0.2423557	Inflammatory bowel disease (IBD)
**ko04662**	9 (0.87%)	102 (0.67%)	0.2484696	B cell receptor signaling pathway
**ko04623**	9 (0.87%)	105 (0.69%)	0.2755423	Cytosolic DNA-sensing pathway

### Identification of cytokines and their expression analysis after different challenges

**Fig. 4:**
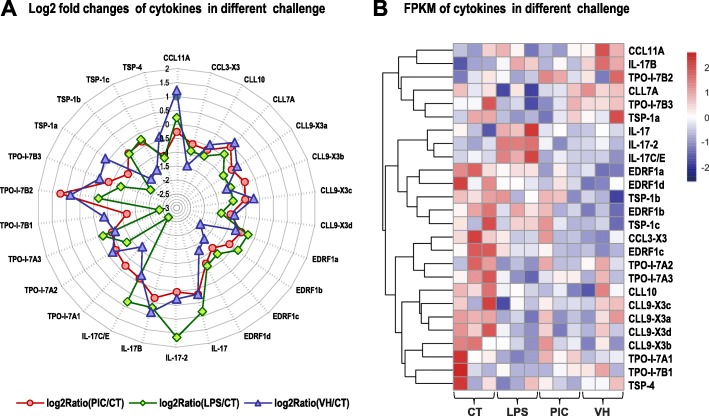
Changes in expression of cytokine genes upon immune challenges of LPS, Poly (I: C) and *V. harveyi.* **a** The periphery of the circle is the X-axis which presents cytokine genes, while the radius of the circle is the Y-axis which presents the log 2 fold changes value of LPS, Poly (I:C) and *V. harveyi* immune challenges of corresponding cytokine genes. The closer from the edge of the circle the higher the up regulated expression of corresponding gene is, and the closer from the center of the circle the higher the down regulated expression of corresponding gene is. **b** two-dimensional hierarchical clustering performed on the clusters of cytokine genes FPKM under three different stimulis. The pink and blue color represents the up- and down-regulation, respectively

Cytokines are a broad and loose category of small proteins (~ 5–20 kDa) that are important for cell signaling, especially the immune signaling. Twenty-one cytokines were selected according to the NR database annotation of the transcriptomic data. The identified cytokines belong to four cytokine families, namely, the B-cell lymphokine (BCL/CLL), erythroid differentiation-related factor 1-like (EPRF1), interleukin 17-like (IL-17) and thrombospondin-like (TSP/TPO) families. In our transcriptome for the sea cucumber *H. leucospilota*, the BCL/CLL family included CCL11A, BCL3-X3, BCL10, CLL7A, CLL9-X3a, CLL9-X3b, CLL9-X3c, and CLL9-X3d; the EPRF1 family included EDRF1a, EDRF1b, EDRF1c, and EDRF1d; the IL-17 family included IL-17, IL-17-2, IL-17B, and IL-17C/E; and the TSP/TPO family included TPO-I-7A1–3, TPO-I-7B1–3, TSP-1a-c, and TSP-4. Among these cytokine families, the expression levels of the four genes in the interleukin-17 family after LPS challenge were significantly higher than those of other cytokines (Fig. 4), indicating that IL-17 was an important family of the cytokines in the immune response of sea cucumber, and its effective mechanism needs to be further investigated.

### Phylogenetic and structural domain analysis of the selected cytokines

**Fig. 5:**
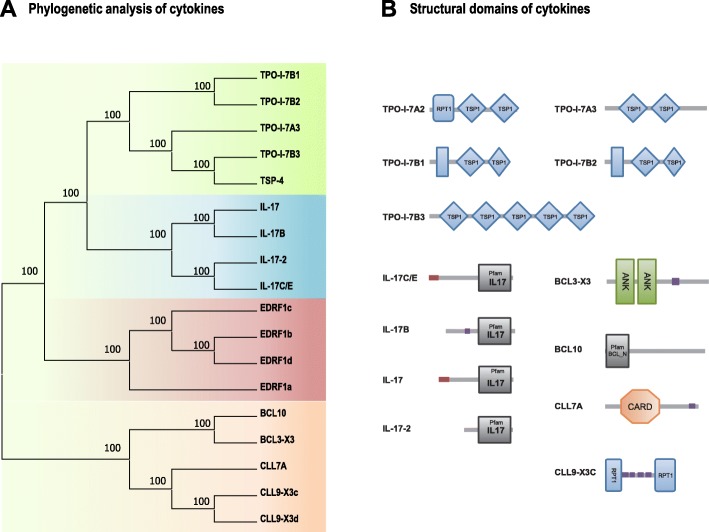
Bioinformatics analysis of selected cytokines. **a** Phylogenetic analysis of the selected cytokines family with maximum-likelihood (ML) method. **b** The structural domains of some of the cytokines

A phylogenetic tree was constructed using a maximum-likelihood (ML) method under MEGA7.0 with the deduced amino acid sequences from the selected cytokines, including BCL/CLL, EPRF1, IL-17 and the TSP/TPO family (Fig. 5a). The result showed that the cytokines were clustered into the corresponding branches.

The structural domains for some of the selected cytokines were predicted and shown in Fig. 5b. Specially, the members of TSP/TPO family contain repeated type 1 thrombospondin domains. The BCL/CLL family cytokines contain a conserved N-terminal domain that is found in the BCL7 family. The predicted IL-17, IL-17-2, IL-17B, and IL-17C/E in the IL-17 family commonly contain a cysteine knot fold domain.

### Transcript expression of cytokines in the coelomocytes with dosage-increased LPS treatment

**Fig. 6:**
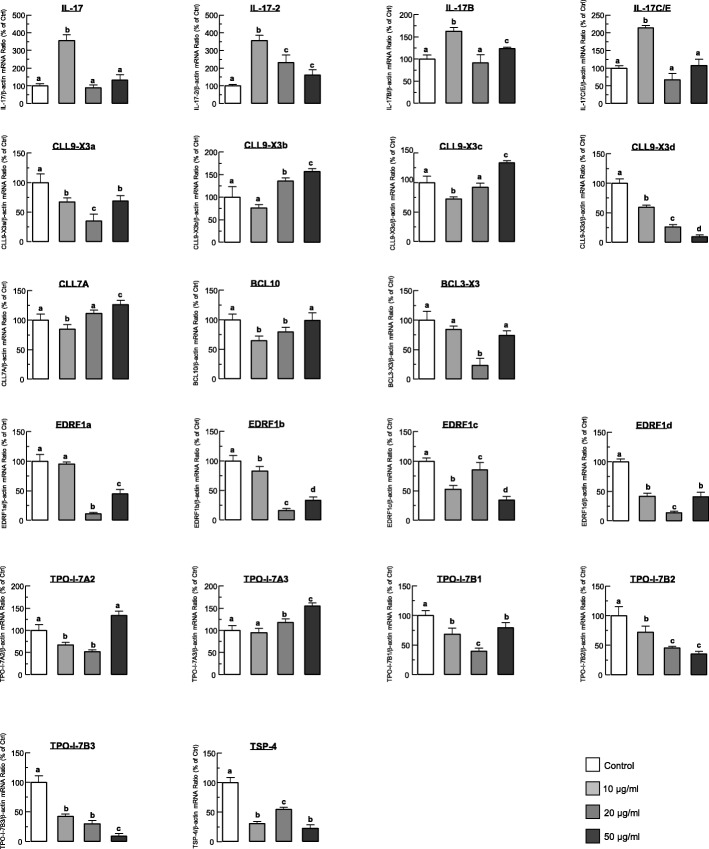
Dose-increased expression of cytokine genes. Transcriptional expression of *H. leucospilota* cytokines gene, with the *β-actin* as reference gene, in the sea cucumber coelomocytes treated respectively with LPS concentration of 10.0 μg/ml, 20.0 μg/ml, 50.0 μg/ml for 24 h. The data presented are expressed as the mean ± S.E. (*n* = 3). The same letter in the experimental groups represent a similar level (*p* > 0.05, ANOVA followed by Fisher’s LSD test)

The selected cytokines were applied to study their expressing upon LPS treatments of increased concentrations of 10.0 μg/ml, 20.0 μg/ml and 50.0 μg/ml. As the result, the mRNA levels of IL-17 cytokines family were significantly upregulated after 10 μg/ml LPS challenge, and their expression levels generally showed a trend of first increasing, then decreasing, and finally stabilizing with the increasing of LPS concentration (Fig. 6). In the BCL/CLL family, with the increase of LPS concentration, BCL10, CLL7A, CLL9-X3b and CLL9-X3c showed a dose-dependent increasing expression pattern, similar to the expression patterns of TPO-I-7A2 and TPO-I-7A2 in the TSP/TPO family. In contrast, CCL11A, BCL3-X3, CLL9-X3a and CLL9-X3d were expressed in a dose-dependent descending pattern, similar to the expression trends of TPO-I-7B1–3 and TSP-4 in the TSP/TPO family. In the EPRF1 family, EDRF1a, EDRF1b, EDRF1c, and EDRF1d showed similar parallel expression patterns, which first increased, then decreased, and finally stabilized with increasing of LPS concentrations (Fig. 6).

## Discussion

Based on Illumina HiSeq2500 sequencing, a transcriptome for the sea cucumber primary coelomocytes under challenges of LPS, Poly (I: C) and *V. harveyi* was obtained in this study. Without referenced genomic data, the assembled Unigene sequences were annotated using the databases of nucleotides and genomes of other species. There are 28.98 and 31.91% of the unigenes that annotated to the NR database were matched to the sequences from *S. purpuratus* and *A. planci*, respectively. One of the possible reason is that the first two reference genomes databases were supplied in these two species [27, 28]. However, other two studies for the genome of the sea cucumber *A. japonicas* have been published recently [19, 29], which can provide more conveniences for annotating the genes in the transcriptome of other sea cucumber speices in the future.

A Unigene in the transcriptomic data is declared differentially expressed if a difference observed in read counts between the experimental and control conditions is statistically significant. Thirteen Unigenes were selected from the DEGs for RT-qPCR verification (Fig. 2c & d). The results showed that the trends of gene expression by RT-qPCR were consistent with those by RNA-seq analysis, verifying the credibility of the RNA- seq results.

The co-expressed DEGs in all three groups with different immune challenge [LPS, Poly (I: C) and *V. harveyi*] may speculated to be the essential genes of the immune defense in sea cucumber, while the DEGs unique to a kind of immune stimuli may specifically respond to the corresponding stimuli. LPS is the main component of the wall of gram-negative bacteria, as a typical endotoxin, can bind to the CD14/TLR4/MD2 complexes in many cell types, especially monocytes, dendritic cells, macrophages and B cells, which can promote the secretion of pro-inflammatory cytokines, nitric oxide and eicosenoic acid, leading to a series of immune responses in the host [30]. In the LPS-treatment group, the Th1 and Th2 cell differentiation pathway was the most significantly enriched immune-related pathway. Poly (I:C), structurally similar to double-stranded RNA [31], is an immune activator that is used to mimic viral infection, and it is known to interact with TLR3, which is expressed on the cell membrane of B cells, macrophages and dendritic cells. In the Poly (I:C) treatment group, the intestinal immune network for IgA production pathway was the most significantly enriched immune-related pathway. *V. harveyi* is a gram-negative marine bacterium that is pathogenic to commercial aquaculture species, including shrimp, fish, and sea cucumber [32]. In the *V. harveyi* treatment group, the Fc gamma R-mediated phagocytosis pathway was the most significantly enriched immune-related pathway. Annotation of the DEGs into the immune-related pathways provides a basis for the research of immune molecular mechanism of echinoderms.

For analysis of DEGs in the coelomocytes with different immune challenges, the results showed that compared with the control group, more down-regulated DEGs than up-regulated DEGs were presented in the groups challenged with three different immune stimuli (Fig. 2b), indicating that the expression for a majority of genes were suppressed by immune challenge. The top 10 significantly enriched KEGG pathways (Tables 2 and 4) and immune-related KEGG pathways (Tables 3 and 5) for common and specific immune challenged DEGs were further screened out. Based on the transcriptomic data in this study, the cDNAs of *H. leucospilota* Fas-associated death domain (FADD) have been cloned with functional characterization [33] It has been reported that there are many humoral immune factors in the coelomic fluid of echinoderms that can recognize and attack the invaded pathogens, including different types of lectin, lysozyme, hemolysin, hydrolase, phenol oxidase, superoxide dismutase, nitric oxide synthase and complement-like factor [6, 9, 34–36]. Similarly, a number of humoral immune factors in the corresponding immune-related pathways (Table 5) were identified in the current study.

The top 10 KEGG pathways for the most significant DEG enrichment after different immune challenges also included amino acid and carbohydrate metabolic pathways, such as alanine, aspartate and glutamate metabolism pathway, amino sugar and nucleotide sugar metabolism pathway, and glycosaminoglycan biosynthesis-heparan sulfate/heparin pathway (Table 4). A similar study performed in in the sea cucumber *A. japonicas* has reported that the arginine metabolic pathway is related to the pathogenic challenge with a dose-dependent manner [37]. Combined with our current study, it is speculated that the metabolism of some amino acids is related to the host immune response in echinoderm, and the regulatory network between immunity and metabolism is more complicated than known now.

Based on the DEGs identified in the transcriptome of sea cucumber coelomocytes challenged with immune stimuli, twenty-one candidate genes of immune cytokines were selected for analyzing the cytokine response of sea cucumber against invaded pathogens. The selected cytokines belong to four cytokine families, namely, the TSP/TPO, BCL/CLL, EPRF1 and IL-17 families (Fig. 5a). Among these gene families, the expression levels of 4 genes in the IL-17 family after LPS challenge were significantly higher than those of other cytokines. By further confirmation with RT- qPCR of the expression levels of 4 IL-17 s generally showed a trend of first increasing, then decreasing, and finally stabilizing with incubation of LPS with increasing concentrations (Fig. 6). IL-17 is a kind of potent proinflammatory cytokine produced by activated memory T cells in mammals [38]. The IL-17 family (of which there are 6 known members, termed IL-17A to IL-17F) is thought to represent a distinct signaling system that appears to highly conserved across vertebrate evolution [38]. IL-17 family members play an active role in inflammatory diseases, autoimmune diseases and cancer [39]. Based on the identification of differentially expressed cytokine genes in the sea cucumber coelomocytes under immune challenges, this study may provide an evidence that many kinds of cytokines interact with each other, and play a complex and essential role in the innate immunity of sea cucumber.

At present, more and more studies on the immune system of the sea cucumber have been developed at both the molecular and cellular levels, which were based on the basal information for immune-related genes that provided by the transcriptomic studies [25]. However, the specific immune defense mechanism in the effector coelomocytes is still unclear. In this study, high-throughput transcriptome sequencing and bioinformatics analysis were performed on coelomocytes treated with three different immune stimuli (LPS, Poly (I:C) and *Vibrio harveyi*). Analysis of the results could elucidate the molecular mechanism for the immune response in the sea cucumber coelomocytes to the pathogenic/immune challenge, laying a foundation for the future identification of immune-related genes and characterizations of immune-responded mechanisms of echinoderm.

## Conclusions

In this study, comparative transcriptome analysis of DEGs among LPS (10 μg/ml), Poly (I:C) (10 μg/ml) and heat-inactivated Vibrio harveyi (10^7^ cells/ml) immune stimuli of sea cucumber H. leucospilota coelomocytes revealed the DEGs and immune-related pathways that are crucial for research of molecular mechanisms related to the immune response in echinoderms. Twenty-one cytokine candidate DEGs were identified, which belong to 4 cytokine families, namely, BCL/CLL, EPRF1, IL-17 and TSP/TPO. Among them IL-17 family cytokines were significantly upregulated after 10 μg/ml LPS challenge for 24 h in response to LPS dose-increased treatment (0, 10, 20 and 50 μg/ml), which provide new insights for the echinoderm cytokine response during immune challenge.

## Methods

### Animals

Animals used in this research were obtained from commercial sea cucumber catches, therefore approval from any ethics committee or institutional review board was not necessary. A total of nine healthy *H. leucospilota* weighing 190–210 g were obtained from the Daya Bay in Guangdong province, China, and maintained in a seawater aquarium with aerated and filtrated seawater (30‰ salinity) at 32 °C for 1 week before the experiments were performed.

### Isolation, primary culture and static incubation of coelomocytes

Given that coelomocytes are considered as the immune effector cells in echinoderms [10], the coelomocytes from *H. leucospilota* were applied to explore the response of sea cucumber after challenge of immune stimuli. Isolation of primary cells and static incubation of coelomocytes were performed as previously reported with some modifications [40, 41]. Briefly, three sea cucumbers were washed with sterile DEPC water three times before dissection on ice. The coelomic fluids were sieved through a 150-mesh cell sieve to remove large tissue debris, mixed with the prechilled anticoagulant solution (20 mM EGTA, 480 mM NaCl, 19 mM KCl and 68 mM Tris, pH 7.6) in a 1:1 volume ratio. The cell suspension was filtered through a 100-μm nylon mesh, and the cells were collected by centrifugation at 500×g at 4 °C for 10 min. The harvested cells were washed twice with 30 ml isotonic buffer (1 mM EGTA, 530 mM NaCl and 10 mM Tris, pH 7.6) and resuspended in 10 ml Leiboviz’s L-15 cell culture medium (Gibco BRL, USA; containing 390 mM NaCl, 100 U/mL penicillin and 100 μg/ml streptomycin, pH 7.6) and filtered through a 40-μm nylon mesh to remove the cell clusters. The cell yield was counted with a 0.10-mm hematocytometer, and only the cell preparations with greater than 95% viability, assessed by trypan blue exclusion assay, were used in subsequent experiments.

The cell suspensions were diluted to 0.1 × 10^6^ cells/ml and seeded onto two 24-well culture plates (1 ml/well) that were precoated with ploy D-lysine. The isolated coelomocytes were incubated at 28 °C for another 18 h for recovery. On the following day, 1 ml of immune stimulus prepared in Leiboviz’s L-15 medium was gently overlaid onto the cells after removal of the old medium. The immune stimuli used in this study included: LPS (10 μg/ml), Poly (I:C) (10 μg/ml) and heat-inactivated *Vibrio harveyi* (10^7^ cells/ml), and fresh Leiboviz’s L-15 medium was used as a control. For RNA sequencing, the cells were harvested at 24 h after incubation of tested substrates. In this case, cells from four wells were pooled together as a biological duplicate, and three duplicates for each group were sequenced and analyzed.

### RNA extraction, library construction and sequencing

Total RNA from each sample was extracted using TRIzol Reagent (Invitrogen, USA). The quality and concentration of total RNA were determined with NanoDrop (Thermo Scientific, USA), and the RNA integrity value (RIN) was checked using the RNA 6000 Pico LabChip on an Agilent 2100 Bianalyzer (Agilent, USA). The RNA-seq library was constructed in six steps: 1) ployA-tailed mRNAs were enriched by oligo (dT) selection; 2) the obtained mRNAs were fragmented and reverse transcribed into double-stranded cDNA (dscDNA) by N6 random primer; 3) the cDNA ends were repaired by 3′-adenylation and adaptor ligation; 4) the ligation products were amplified by PCR; 5) the PCR products were heat-denatured and the single-stranded DNAs were cyclized; and 6) sequencings were performed on an Illumina HiSeq2500 (Illumina, USA).

### De novo assembly and Unigene functional annotation

The raw sequencing data were first filtered by using SOAPnuke (v1.5.2) software under the parameters of -l 15 -q 0.2 -n 0.1 to remove the reads in which unknown bases (N) are more than 5%. The low quality reads, which were defined by the base qualities for more than 20% of them were less than 15, were then removed. After reads filtering, de novo assembly was performed using Trinity2.0.6 with clean reads [42]. Tgicl2.0.6 was used on cluster transcripts to remove redundancy and get Unigenes [43]. After assembly, the Unigenes functional annotation were performed with seven functional databases, namely NR, NT, GO, KOG, KEGG, SwissProt and InterPro. Specifically, We use Blastn 2.2.23 [44], We use Blastx 2.2.23 [44] or Diamond 0.8.31 [45] align Unigenes to NT, NR, KOG, KEGG and SwissProt database to do the annotation, use Blast2GO 2.5.0 [46] with NR annotation to do the GO annotation, and use InterProScan5 5.11–51.0 [47] to do the InterPro annotation, under default parameter.

### Quantification of gene expression level and identification of DEGs

Clean reads of each sample were mapped to the Unigenes with Bowtie2 2.2.5 [48], and the gene expression levels were calculated with RSEM 1.2.12 (RNA-Seq by Expectation-Maximization) to obtain the FPKM (Fragments Per Kilobase of exon model per Million mapped reads) value [49]. The DEGseq algorithm was used to detect the differences of gene expression between different groups. To improve the accuracy of DEGs, genes with over two-fold changes (log2 ratio ≥ 1) and q-value < 0.001 were considered as significantly DEGs.

### Screening and analysis of immune-related pathways

Based on Wallenius non-central hyper-geometric distribution [50], GO enrichment analysis of the differentially expressed genes (DEGs) was implemented by the GOseqR packages 1.10.0, which can be adjusted for gene length bias in DEGs. KOBAS (KEGG Orthology-Based AnnotationSystem) software was used to test the statistical enrichment of DEGs in KEGG pathways. The FDR correction was performed for *p*-value, and pathways with FDR < 0.01 were regarded as significantly enriched pathways. The top 10 immune-related pathways significantly enriched in the groups treated with different stimuli were selected by the immune-related annotation of the KEGG pathways.

### Validation of differentially expressed genes by RT-qPCR

Significant DEGs (*P* < 0.05, FDR < 0.05, fold change ≥8) were selected for RT-qPCR analysis to validate the transcriptomic data, and corresponding primers were designed by based on the Unigenes sequences (Table 1). The primary coelomocytes isolated from another three *H. leucospilotas* were then challenge by LPS (10 μg/ml) for 24 h. Total RNA from each sample was extracted using TRIzol Reagent and reverse transcribed into the first cDNA using Honorll 1st strand cDNA Synthesis SuperMix for qPCR kit (Novogene, China). Finally, quantitative real-time PCRs were performed using the Unique Aptamer qPCR SYBR probe kit (Novogene). In this case, the qPCRs were performed in triplicates for cells cultured in three individual wells.

### Identification and bioinformatics analysis of cytokines responding to immune challenge

According to the NR annotation of the transcriptomic data from the coelomocytes challenged by LPS, Poly (I:C) and *V. harveyi*, Twenty-one candidate cytokine genes were selected. The log2-fold changes of their expression levels under different challenges were presented as heatmap by the Pheatmap package using R3.5 software [51, 52].

The CDSs of the candidate cytokine genes were translated to amino acid sequences to construct a maximum-likelihood phylogenetic tree with 1000 bootstrap replicates by using MEGA 7. The structural domains of the cytokines were predicted by using the SMART program.

### Measurement of cytokines mRNA levels in coelomocytes after LPS challenge

Primers for the selected cytokine genes were designed by based on their Unigenes sequences (Table 6). The primary coelomocytes isolated from the other three *H. leucospilotas* were obtained after treatment of LPS in gradient increased concentrations (0, 10, 20 and 50 μg/ml) for 24 h. Total RNA extraction, first-strand cDNA synthesis and RT-qPCR were performed as described above. In this case, β-actin was used as a reference gene, and the RT-qPCRs were performed in triplicates for cells cultured in three individual wells.

**Table 6:** Primers designed for RT-qPCR of DEGs

Gene ID	***P***-values	Forward primer sequences (5′-3′)	Reverse primer sequences (5′-3′)
**Differentially expressed genes(DEGs)**			
**CL3876.Contig4_Mix**	0	TCACGGCATTGAGAACGG	CGGTGTCTGAACTTTGGGT
**Unigene38924_Mix**	0	CAGACAAAAGCCTCAGAAATG	AAGAACCCAGAGCTGGAAAG
**Unigene36248_Mix**	0	ATACGAAAAGCGGCAAACA	TCCATCTCAGACCCCAACTC
**Unigene1474_Mix**	0	TCCATCTCAGACCCCAACTC	TTTCCATTGGGAGGTAAGC
**Unigene6464_Mix**	0	TCACTGCGGCATTGATTC	GGTGCCCTTCCGTCAATT
**Unigene36250_Mix**	0	GTGCAAAGAAGGGAAGGC	TCTGGTGATTTATGTTGTGGG
**Unigene42438_Mix**	0	ACTCATGTGGATGGCACTAGC	GGAAGAAGGGACAGAATAAACC
**CL3998.Contig1_Mix**	1.89E-260	AAGGATTATGGACGGGACAC	TTCTCAAACCACCAGAGGG
**Unigene1910_Mix**	0	GTCAAACCAATAACAGCGAAAG	TGGACCAGACCAGGCAAA
**Unigene27584_Mix**	0	CGTGCTTGCCGAGTTTGT	GCAGGCTATTGCTGTTGTTAA
**CL657.Contig6_Mix**	0	AAGCAAGATAGACACTGTGGTTC	GATTGATGCCCCGTAAGC
**CL3619.Contig1_Mix**	0	AGGGTAACAGGTTGGGTGAA	GCAGTAGTTGGAGCCATTGAA
**Unigene11374_Mix**	0	TGCGTTTGTTGACTTTTGC	TGAAATGGTTCCGATTCTCA
**Unigene2830_Mix**	0	TTTGCTGGAGTATTGTGGTTC	TGGGCTGTAGGGGCTTTA
**Unigene6841_Mix**	2.03E-139	ACCCCAGATGACCTTTGACC	GTTAACACAATTTCTCCAGTTAGGA
**Unigene2638_Mix**	1.05E-297	AGTTGATATTCACCAGCCTTGC	CCACTTTCCATGAAACCGTAA
**Unigene1366_Mix**	2.56E-93	GGAGGTCAATGGTCATTTGG	ACCCCTGGTGACCCTACAT
**Unigene1518_Mix**	7.31E-233	AAACACGGTATCTCCAGAATGA	AATGACCTTTGACCTCCACC
**Unigene16828_Mix**	0	TTCAAAAGCAGTGCCAACG	CATTATCATCATCTTGCCGAAC
**Unigene3853_Mix**	2.50E-62	CCACTTGGACAGCAGTGAAG	TGATGACCTGGTTTGAGAAGAC
**Cytokine genes**			
**CCL3-X3**	0.000883536	CGCAAAGGATTGTGATGGT	ACAGGAAGAGGCTATACAGACG
**CLL10**	0.013152297	CTTACTGGGTTGGTAGTTTTGAC	GTGCGTTCTTGCCATTCTC
**CLL7A**	0.00023473	TCTTGCTTCTCACCCGATTT	CCGTAGGGGACACCTCTTT
**CLL9-X3a**	5.57E-09	TGGTGGAATAGTGGAGGGTG	GACTGGTTGGTTGAGGCTGT
**CLL9-X3b**	0.003436624	AACAGACGGACAGTCATACAGC	TGGTTGAGCCCTTGATAGTG
**CLL9-X3c**	0.005767319	CATTCCAGTTGGTGATGGC	TGATTTCCTCACCGCATTT
**CLL9-X3d**	2.23E-07	CAGAAAGCCTGGGTAAAGC	GACCTGGACCAAACCTCATT
**EDRF1a**	0.101229003	TTACAAGACTATCCTCCCCTCA	GCGATGCGATACTGATAAAGG
**EDRF1b**	0.311031428	ATGGCTTTTGAGGATGGTG	GCTGCTGTATCTCAGGAGGAAA
**EDRF1c**	0.000470648	ACCAACCACCATCCAACTG	TTCTCCTGATCCACCCCTAA
**EDRF1d**	0.001415506	TCCCTTTATTGTTCCTGTGG	TGTCTCCGCTTTCCTGTAGTA
**IL-17**	1.94E-07	AGGCTGGCTCGCTTCTT	ACGCTATCGCTCATTGGAC
**IL-17-2**	5.40E-08	GTTGAGGCATTCACATTTCG	AACTTTCGGCAGTCCTTACAG
**IL-17B**	1.36E-05	TACCATAGGAAGGGGATCTGT	AGACTTGCCCGACGATTT
**IL-17C/E**	3.56E-23	AGGCACTCTTCTGCATTCTCC	GCTACTGAACGCTTGTTGACG
**TPO-I-7A2**	1.90E-07	CGGCGGCATACAACAGA	ACATTGGGCATCCAGAACA
**TPO-I-7A3**	0.003209029	GGTCCAAAGAAAGGCATCA	GGCTTGAAAGTAAGAAACCGT
**TPO-I-7B1**	5.20E-08	GATTCAGCCTTCATTTTGGA	GCTTAGTATCTTTGGGTCTGTCTA
**TPO-I-7B2**	0.000343861	GATTCAGCCTTCATTTTGGA	GCTTAGTATCTTTGGGTCTGTCTA
**TPO-I-7B3**	0.7252968	TGGTCCAAGTGGTCTCCCT	CTGGTCCTCTGGTAAACAGTCAT
**TSP-4**	0.086692271	CAGGAGTCTTATTTGGTATCTATGC	CAATGTGGCGATGGTGTTC

## Data Availability

RNA-seq data from this article have been deposited in NCBI under the accession number of STUDY: PRJNA559679, SAMPLE: D2CT (SAMN12551561), EXPERIMENT: C1_1 (SRX6693868), RUN: D2CT1_1.fq.gz (SRR9945372), SAMPLE: D2LPS (SAMN12551562), EXPERIMENT: L2_2 (SRX6693869), RUN: D2LPS1_1.fq.gz (SRR9945371), SAMPLE: D2PIC (SAMN12551563), EXPERIMENT: P3_3 (SRX6693870), RUN: D2PIC1_1.fq.gz (SRR9945370), SAMPLE: D2VH (SAMN12551564), EXPERIMENT: V4_4 (SRX6693871), RUN: D2VH1_1.fq.gz (SRR9945369).

## Abbreviations

ANOVA: Analysis of variance
BCL/CLL: B-cell lymphokine
CT: Control group
DEG: Differentially expressed genes
EPRF1: Erythroid differentiation-related factor 1
FPKM: Fragments per kilobase of exon model per million mapped reads
GO: Gene ontology
GSEA: Gene set enrichment analysis
IL-17: Interleukin 17
KEGG: Kyoto encyclopedia of genes and genomes
KOG: Clusters of orthologous groups for eukaryotic complete genomes
LPS: Lipopolysaccharide
MAPK: Mitogen-activated protein kinase
ML: Maximum-likelihood
NGS: Next-generation sequencing
NR: Nonredundant
PIC: Polyinosinic:polycytidylic acid
Poly (I:C): Polyinosinic-polycytidylic acid
RIN: RNA integrity value
RSEM: RNA-seq by expectation-maximization
RT-qPCR: Real time quantitative polymerase chain reaction
TSP/TPO: Thrombospondin
VH: *Vibrio harveyi*
